# Supplementary material for: Cardio-centric hemodynamic management improves spinal cord oxygenation and mitigates hemorrhage in acute spinal cord injury
Source: Nat Commun. 2020 Oct 15;11:5209. doi: 10.1038/s41467-020-18905-8 (PMC7562705; doi:10.1038/s41467-020-18905-8)
Supplement: Supplementary file 1 — Supplementary Information [file 41467_2020_18905_MOESM1_ESM.pdf]

Cardio-centric hemodynamic management improves spinal cord oxygenation and mitigates hemorrhage in acute spinal cord injury

Williams et al.

Supplementary Information

Supplementary Table 1. Experiment 1: left ventricular load-dependent pressure-volume indices, and systemic hemodynamics

		PRE-SCI	POST-SCI			
	**RM-ANOVA**	***Baseline***	***1 h***	***2 h***	***3 h***	***4 h***
		**LV volumetric measures**				
**EDV** (ml)	**p=0.003**	57.7 (9.9)	56.5 (10.6)	58.0 (11.4)	60.6 (11.0)	62.4 (13.7)**
**ESV** (ml)	**p=0.04**	32.3 (6.9)	30.2 (6.3)	30.7 (6.4)	31.1 (5.6)	33.4 (7.2)
**SV** (ml)	p=0.10	27.4 (5.9)	28.0 (5.9)	29.0 (6.1)	31.3 (6.4)	30.7 (8.5)
**EF** (%)	p=0.27	49.5 (8.2)	52.4 (6.8)	52.8 (7.0)	54.5 (7.5)	52.3 (10.1)
		**LV systolic pressure measures**				
$\mathbf{P_{max}}$ (mmHg)	**p=0.003**	101.0 (12.7)	95.2 (11.0)**	93.2 (10.4)***	93.4 (9.0)***	94.3 (10.2)**
$\mathbf{P_{es}}$ (mmHg)	**p<0.001**	97.2 (15.9)	88.0 (15.2)**	85.5 (13.9)***	85.7 (12.7)***	87.1 (14.3)***
$\mathbf{dp/dt_{max}}$ (mmHg/s)	p=0.24	1630 (194)	1705 (156)	1695 (179)	1685 (168)	1716 (215)
**Stroke work** (mmHg·ml)	p=0.24	2053 (387)	2084 (561)	2114 (560)	2291 (578)	2275 (721)
$\mathbf{E_a}$ (mmHg/ml)	**p=0.005**	3.69 (1.09)	3.17 (0.94)	3.00 (0.90)*	2.77 (0.81)**	2.94 (2.94)**
		**LV diastolic pressure measures**				
$\mathbf{P_{ed}}$ (mmHg)	p=0.89	15.3 (3.7)	14.6 (3.5)	14.9 (3.5)	14.6 (2.6)	15.2 (3.2)
$\mathbf{dp/dt_{min}}$ (mmHg/s)	p=0.15	-2357 (696)	-2223 (662)	-2182 (709)	-2150 (668)	-2212 (650)
**τ** (ms)	p=0.93	43 (7)	42 (7)	43 (8)	42 (7)	42 (6)
		**Systemic and pulmonary hemodynamics**				
**MAP** (mmHg)	**p<0.001**	85.4 (13.2)	79.5 (12.5)**	77.4 (12.6)***	78.6 (11.2)***	78.9 (11.4)***
**SBP** (mmHg)	**p=0.012**	116.2 (12.5)	111.1 (15.1)**	110.0 (15.2)***	112.3 (13.5)*	112.7 (13.9)*
**DBP** (mmHg)	**p<0.001**	69.9 (14.3)	63.6 (12.1)**	61.1 (12.3)***	61.7 (11.1)***	62.0 (11.4)***
$\mathbf{Q_{TD}}$ (ml/min)	p=0.07	2685 (473)	2902 (463)	2528 (1126)	2528 (1080)	2572 (1085)
**HR** (bpm)	p=0.45	89.3 (19.0)	94.6 (15.1)	92.9 (14.3)	91.1 (15.0)	92.4 (14.0)
**TPR** (mmHg/L/min)	**p=0.003**	37.4 (10.6)	31.2 (6.8)**	29.8 (6.8)**	28.9 (7.3)***	29.9 (9.5)**
**mPAP** (mmHg)	p=0.15	21.1 (4.1)	22.6 (4.7)	22.6 (4.3)	22.4 (3.6)	22.6 (3.7)
**PASP** (mmHg)	p=0.12	31.5 (4.3)	32.8 (5.3)	33.0 (5.0)	33.0 (4.5)	32.9 (4.8)

Values are means (SD). Time effects shown for one-way repeated measures ANOVA (RM-ANOVA), pairwise comparisons versus pre-SCI (baseline) performed using Fisher's LSD. LV: left ventricular; EDV: end-diastolic volume; ESV: end-systolic volume; SV: stroke volume; EF: ejection fraction; P_{max}: maximal systolic pressure; P_{es}: end-systolic pressure; dp/dt_{max}: maximal rate of pressure generation; E_a: arterial elastance; P_{ed}: end-diastolic pressure; dp/dt_{min}: maximum rate of pressure decay; **τ**: rate constant of pressure decay; MAP: mean arterial pressure; SBP: systolic blood pressure; DBP: diastolic blood pressure; Q_{TD}: cardiac output assessed with thermodilution; HR: heart rate; TPR: total peripheral resistance; mPAP: mean pulmonary arterial pressure; PASP: pulmonary arterial systolic pressure. *p<0.05 vs pre-SCI; **p<0.01 vs pre-SCI; ***p<0.001 vs pre-SCI.

Supplementary Table 2. Experiment 1: left ventricular load-independent function and ventricular-vascular coupling

		PRE-SCI	POST-SCI			
	**RM-ANOVA**	***Baseline***	***1 h***	***2 h***	***3 h***	***4 h***
**E_{es}** (mmHg/ml)	**p<0.001**	2.66 (0.76)	2.16 (0.72)***	2.17 (0.73)***	2.08 (0.62)***	2.11 (0.70)***
**$EDPVR_{B1}$**	p=0.24	0.018 (0.008)	0.014 (0.008)	0.017 (0.007)	0.015 (0.006)	0.015 (0.007)
**PRSW** (mmHg)	**p=0.013**	57.2 (13.8)	46.7 (12.4)**	48.2 (13.1)*	47.0 (9.5)***	46.8 (11.7)***
**dp/dt_{max}-EDV** (mmHg/s/ml)	**p<0.001**	29.3 (12.5)	21.5 (12.2)**	23.3 (13.5)**	18.8 (9.9)***	17.7 (9.6)***
**E_a/E_{es}**	p=0.58	1.42 (0.39)	1.52 (0.41)	1.43 (0.37)	1.36 (0.35)	1.43 (0.44)

Values are means (SD). Statistics are identical to those in Supplementary Table 1. E_{es}: end-systolic elastance, index of contractility; $EDPVR_{B1}$: end-diastolic pressure-volume relationship slope; PRSW: preload-recruitable stroke work; dp/dt_{max}-EDV: maximal rate of pressure generation for a given end-diastolic volume; E_a/E_{es}: ventricular-vascular coupling, arterial elastance-to-end-systolic elastance. *p<0.05 vs pre-SCI; **p<0.01 vs pre-SCI; ***p<0.001 vs pre-SCI.

Supplementary Table 3. Experiment 2: left ventricular load-independent function and ventricular-vascular coupling

	RM-ANOVA EFFECTS			GROUP	PRE-SCI	POST-SCI				
	time	*group*	*gr × time*		*Baseline*	*30 min (pre-drug)*	*1 h*	*2 h*	*3 h*	*4 h*
**E_{es}**	p=0.098	**p<0.001**	**p<0.001**	CON	2.66 (0.76)	-	2.16 (0.72)	2.17 (0.73)	2.08 (0.62)	2.11 (0.70)
(mmHg/ml)				DOB+ †	1.93 (0.63)	1.90 (0.45)	3.13 (0.90)*	3.26 (1.26)*	3.54 (1.48)*	4.20 (1.96)**
				DOB- †	3.36 (1.64)	2.15 (0.30)	3.61 (1.02)**	3.24 (0.14)*	3.05 (0.19)	3.34 (0.16)*
				NE ††	2.26 (0.94)	2.21 (0.61)	4.39 (1.70)** [a]	4.99 (2.35)*** [a]	4.83 (2.27)** [a]	4.55 (1.92)*** [a]
**$EDPVR_{B1}$**	p=0.17	p=0.80	p=0.56	CON	0.012 (0.009)	-	0.021 (0.014)	0.020 (0.014)	0.020 (0.017)	0.020 (0.018)
				DOB+	0.019 (0.009)	0.026 (0.019)	0.023 (0.011)	0.021 (0.009)	0.020 (0.007)	0.020 (0.009)
				DOB-	0.027 (0.017)	0.031 (0.011)	0.034 (0.017)	0.030 (0.011)	0.033 (0.014)	0.023 (0.005)
				NE	0.022 (0.008)	0.023 (0.012)	0.028 (0.011)	0.027 (0.012)	0.024 (0.009)	0.025 (0.008)
**PRSW**	p=0.055	**p<0.001**	**p<0.001**	CON	29.3 (12.6)	-	21.5 (12.2)	23.3 (13.5)	18.8 (9.9)	17.7 (9.6)
(mmHg)				DOB+ †††	15.4 (8.6)	20.4 (6.6)	49.5 (14.8)***	50.5 (26.9)*** [a]	58.5 (4.0)*** [a]	59.4 (10.7)*** [a]
				DOB- ††	36.6 (7.2)	36.0 (5.6)	49.2 (7.0)**	50.4 (5.3)**	43.4 (6.7)**	45.3 (7.6)**
				NE †††	25.4 (13.1)	24.8 (20.3)	52.9 (28.3)*** [a]	77.2 (54.8)*** [a]	101.6(120.9)*** [a]	99.9 (99.5)*** [a]
**dp/dt_{max}-EDV**	p=0.097	**p<0.001**	**p<0.001**	CON	57.2 (13.8)	-	46.7 (12.4)	48.2 (13.1)	47.0 (9.5)	46.8 (11.7)
(mmHg/s/ml)				DOB+ †	38.4 (15.3)	38.5 (11.9)	75.8 (5.5)**	81.2 (4.8)**	83.9 (7.5)** [a]	83.3 (11.5)** [a]
				DOB- †††	59.9 (20.0)	48.4 (7.6)	66.3 (15.8)***	68.6 (8.1)***	66.7 (14.9)**	66.6 (15.6)***
				NE †††	48.8 (18.7)	46.7 (21.4)	78.9 (24.7)** [a]	78.5 (20.2)***	84.9 (28.5)*** [a]	88.2 (26.3)*** [a]
**E_a/E_{es}**	p=0.41	**p<0.001**	**p<0.001**	CON	1.42 (0.39)	-	1.52 (0.41)	1.43 (0.37)	1.36 (0.35)	1.43 (0.44)
				DOB+ ††	1.91 (0.57)	1.62 (0.51)	0.89 (0.30)***	0.91 (0.34)*** [a]	0.87 (0.29)***	0.78 (0.45)*** [a]
				DOB- ††	1.27 (0.58)	1.36 (0.15)	0.85 (0.20)***	0.88 (0.06)** [a]	0.98 (0.12)**	0.89 (0.04)**
				NE ††	1.74 (0.87)	1.57 (0.59)	0.96 (0.69)**	0.96 (0.79)**	0.95 (0.72)***	0.94 (0.76)***

Values are means (SD). CON: control (*n*=8); DOB+: high-dose dobutamine (i.e. ≥2.5 µg/kg/min; *n*=4); DOB-: low-dose dobutamine (i.e. ≤0.5 µg/kg/min; *n*=3); NE: norepinephrine (*n*=7). Effects from 2-way RM-ANOVA are shown for time, group and interaction (group x time). See Supplementary Tables 1 and 2 for additional abbreviations. Significant effects for 1-way RM-ANOVA are shown in "GROUP" column with symbols. Post-hoc comparisons were performed using Tukey's HSD for between-group comparisons, and Fisher's LSD for planned within-group comparisons (versus 30 min, pre-drug). One-way RM-ANOVA effect of time from 30 min to 4 h post-SCI: †p<0.05; ††p<0.01; †††p<0.001. Between-group comparisons: [a] p<0.05 vs CON; [b] p<0.05 vs DOB - ; [c] p<0.05 vs NE. Within-group comparisons to 30 min (pre-drug): *p<0.05; **p<0.01; ***p<0.001.

Supplementary Table 4. Experiment 2: left ventricular load-dependent pressure-volume indices for systolic and diastolic function

	**RM-ANOVA EFFECTS**			**GROUP**	**PRE-SCI**		**POST-SCI**			
	time	***group***	***gr × time***		***Baseline***	***30 min (pre-drug)***	***1 h***	***2 h***	***3 h***	***4 h***
					**LV volumetric measures**					
**EDV**	p=0.62	**p=0.006**	**p=0.003**	CON †††	57.7 (9.9)	54.1 (8.6)	56.5 (10.6)	58.0 (11.4)*	60.6 (11.0)***	62.4 (13.7)***
(ml)				DOB+ ††	63.8 (8.2)	65.3 (7.5)	58.2 (7.1)**	58.0 (6.1)**	60.4 (4.6)*	61.6 (5.2)*
				DOB-	52.8 (3.3)	52.5 (2.0)	48.6 (3.8)	51.6 (2.7)	54.9 (4.6)	56.6 (5.2)
				NE	61.4 (10.3)	60.7 (12.1)	54.9 (9.6)	50.1 (11.1)	53.4 (12.1)	54.8 (12.9)
**ESV**	p=0.57	**p<0.001**	**p<0.001**	CON ††	32.3 (6.9)	28.6 (4.8)	30.2 (6.3)	30.7 (6.4)*	31.1 (5.6)*	33.4 (7.2)***
(ml)				DOB+ †††	36.8 (9.3)	36.1 (8.1)	22.7 (5.3)***	21.9 (6.8)***	22.9 (5.4)***	22.6 (4.7)***
				DOB- †	30.8 (5.5)	28.3 (5.5)	22.9 (6.6)**	24.7 (6.6)*	27.5 (7.9)	28.3 (8.3)
				NE †	36.7 (12.0)	34.8 (12.5)	26.0 (8.7)*	23.6 (8.7)**	25.6 (10.7)**	26.1 (11.4)*
**SV**	p=0.58	**p<0.001**	p=0.11	CON	27.4 (5.9)	27.8 (5.7)	28.0 (5.9)	29.0 (6.1)	31.3 (6.4)	30.7 (8.5)
(ml)				DOB+ †††	28.7 (1.7)	31.4 (1.8)	37.2 (2.4)***[a]	37.6 (2.8)***[c]	38.8 (1.2)***[c]	40.3 (2.7)***
				DOB- †	24.6 (2.3)	27.4 (2.6)	29.6 (3.9)	30.1 (2.5)*	30.8 (2.8)**	31.9 (3.0)**
				NE	27.6 (3.8)	28.5 (28.5)	30.6 (4.4)	28.0 (5.2)	29.8 (4.4)	30.9 (4.7)
**EF**	p=0.23	**p<0.001**	**p<0.001**	CON	49.5 (8.2)	52.1 (6.1)	52.4 (6.8)	52.8 (7.0)	54.5 (7.5)	52.3 (10.1)
(%)				DOB+ †††	46.5 (7.2)	49.4 (6.6)	65.2 (4.3)***	66.4 (8.1)***	65.7 (5.8)***	66.9 (5.0)***
				DOB- ††	46.0 (6.9)	51.2 (6.5)	59.2 (7.6)***	57.0 (7.5)***	55.4 (8.3)**	55.6 (8.4)**
				NE ††	49.1 (9.1)	53.1 (7.6)	60.2 (11.7)***	59.5 (10.0)**	59.1 (10.8)**	59.4 (10.7)**
					**LV systolic pressure measures**					
**P_{max}**	**p=0.01**	**p<0.001**	**p<0.001**	CON	101.0 (12.7)	83.0 (11.3)	88.0 (15.2)	85.5 (13.9)	85.7 (12.7)	87.1 (14.3)
(mmHg)				DOB+ †††	103.7 (8.0)	90.9 (6.8)	96.6 (11.9)**	99.2 (11.7)*** [a]	106.9 (10.9)*** [ab]	106.3 (15.3)*** [a]
				DOB- †	95.1 (4.4)	79.4 (11.7)	86.3 (4.8)*	85.7 (4.5)	90.9 (1.9)*	94.6 (4.1)**
				NE †††	97.1 (12.8)	84.3 (16.9)	101.1 (11.2)*** [a]	97.5 (15.2)*** [a]	101.5 (16.6)*** [a]	99.1 (18.0)**
**P_{es}**	p=0.22	**p<0.001**	**p=0.008**	CON	97.2 (15.9)	91.5 (8.6)	95.2 (11.0)	93.2 (10.4)	93.4 (9.0)	94.3 (10.2)
(mmHg)				DOB+ †	97.9 (7.9)	99.0 (4.2)	108.3 (8.3)	111.1 (7.4)	116.3 (7.4)**	116.6 (11.7)**
				DOB-	89.4 (6.6)	88.1 (8.3)	95.1 (5.6)	93.8 (5.0)	96.0 (2.8)	98.7 (4.1)
				NE †††	91.8 (14.2)	92.7 (14.2)	109.5 (7.8)***	108.1 (8.4)***	110.6 (8.5)***	108.3 (9.5)***
**dp/dt_{max}**	**p=0.004**	**p<0.001**	**p<0.001**	CON	1630 (194)	1731 (235)	1705 (156)	1695 (179)	1685 (168)	1716 (215)
(mmHg/s)				DOB+ †††	1696 (354)	1683 (345)	3168 (726)*** [a]	3523 (615)*** [a]	3722 (241)***	3652 (373)*** [a]
				DOB- ††	1739 (111)	1729 (235)	2540 (551)***	2278 (504)**	2178 (511)*	2337 (621)**
				NE ††	1675 (238)	1667 (195)	3042 (752)** [a]	3508 (1316)*** [a]	3693 (2081)*** [a]	3551 (1769)*** [a]

Supplementary Table 4 *continued.* Experiment 2: left ventricular load-dependent pressure-volume indices for systolic and diastolic function

	RM-ANOVA EFFECTS			GROUP	PRE-SCI	POST-SCI				
	time	*group*	*gr × time*		*Baseline*	*30 min (pre-drug)*	*1 h*	*2 h*	*3 h*	*4 h*
**Stroke work**	**p=0.002**	**p<0.001**	**p=0.004**	CON	2053 (387)	1969 (520)	2084 (561)	2114 (560)	2291 (578)	2275 (721)
(mmHg·ml)				DOB+ †††	2351 (189)	2428 (107)	3287 (280)*** [a]	3401 (99)*** [abc]	3649 (86)*** [abc]	3751 (166)*** [abc]
				DOB- †	1954 (46)	1963 (288)	2335 (317)*	2340 (219)*	2413 (268)**	2547 (354)**
				NE ††	2078 (307)	2111 (271)	2706 (618)**	2464 (433)*	2642 (351)**	2695 (368)**
**E_a**	p=0.73	p=0.95	**p=0.029**	CON	3.69 (1.09)	3.11 (0.81)	3.17 (0.94)	3.00 (0.90)	2.77 (0.81)	2.94 (2.94)
(mmHg/ml)				DOB+	3.43 (0.42)	2.91 (0.28)	2.62 (0.51)	2.66 (0.45)	2.76 (0.32)	2.67 (0.56)
				DOB-	3.67 (0.54)	2.89 (0.23)	2.93 (0.21)	2.86 (0.10)	2.97 (0.24)	2.97 (0.18)
				NE ††	3.23 (0.72)	2.84 (0.99)	3.29 (0.80)**	3.45 (0.93)***	3.36 (0.74)**	3.20 (0.84)*
**LV diastolic pressure measures**										
**P_{ed}**	p=0.25	**p=0.001**	**p=0.028**	CON	15.3 (3.7)	13.3 (2.7)	14.6 (3.5)	14.9 (3.5)	14.6 (2.6)	15.2 (3.2)
(mmHg)				DOB+	14.0 (4.3)	15.0 (4.1)	13.5 (5.9)	13.4 (3.5)	14.9 (5.1)	15.0 (5.3)
				DOB- †	10.4 (4.2)	10.1 (4.0)	8.8 (5.6)	9.6 (4.1)	11.5 (3.0)	13.4 (2.3)*
				NE	13.8 (2.8)	13.4 (3.0)	15.0 (4.9)	13.6 (4.9)	14.6 (5.3)	14.5 (5.3)
**dp/dt_{min}**	**p=0.036**	**p<0.001**	**p=0.009**	CON †	-2357 (696)	-2045 (596)	-2223 (662)**	-2182 (709)*	-2150 (668)*	-2212 (650)**
(mmHg/s)				DOB+ ††	-3152 (706)	-2597 (425)	-2988 (533)	-3311 (654)*	-3596 (760)**	-3482 (1022)**
				DOB- †	-3433 (155)	-2775 (455)	-3426 (136)*	-3434 (160)*	-3328 (258)*	-3435 (139)*
				NE †	-3277 (1073)	-2789 (911)	-3155 (864)*	-3121 (975)	-3406 (988)***	-3303 (1015)**
**τ**	p=0.07	**p=0.046**	**p=0.026**	CON	43 (7)	42 (6)	42 (7)	43 (8)	42 (7)	42 (6)
(ms)				DOB+ †	34 (5)	36 (6)	30 (4)**	30 (2)**	31 (2)**	32 (3)*
				DOB- †††	32 (5)	33 (4)	31 (6)*	32 (6)	36 (6)*	36 (5)**
				NE	39 (5)	41 (7)	41 (10)	40 (11)	39 (10)	42 (8)

Values are means (SD). CON: control (*n*=8); DOB+: high-dose dobutamine (i.e. ≥2.5 µg/kg/min; *n*=4); DOB-: low-dose dobutamine (i.e. ≤0.5 µg/kg/min; *n*=3); NE: norepinephrine (*n*=7). Statistics are identical to those outlined in Supplementary Table 3. See Supplementary Tables 1 and 2 for abbreviations. One-way RM-ANOVA effect of time from 30 min to 4 h post-SCI: †p<0.05; ††p<0.01; †††p<0.001. Between-group comparisons: [a] p<0.05 vs CON; [b] p<0.05 vs DOB - ; [c] p<0.05 vs NE. Within-group comparisons to 30 min (pre-drug): *p<0.05; **p<0.01; ***p<0.001.

Supplementary Table 5. Experiment 2: systemic and pulmonary hemodynamics

	RM-ANOVA EFFECTS			GROUP	PRE-SCI	POST-SCI				
	time	*group*	*gr × time*		*Baseline*	*30 min (pre-drug)*	*1 h*	*2 h*	*3 h*	*4 h*
				**Systemic blood pressure and vascular resistance measures**						
**MAP**	p=0.07	**p<0.001**	**p<0.001**	CON	85.4 (13.2)	77.1 (11.4)	79.5 (12.5)	77.4 (12.6)	78.6 (11.2)	78.9 (11.4)
(mmHg)				DOB+ †††	88.1 (7.8)	82.6 (2.7)	91.5 (3.8)**	94.7 (5.0)*** **a**	100.3 (1.9)*** **a**	99.1 (6.2)*** **a**
				DOB- †	80.4 (5.5)	75.8 (2.2)	79.7 (2.4)	79.2 (2.1)	81.3 (3.0)*	82.4 (3.0)**
				NE †††	76.3 (11.7)	72.7 (12.9)	88.2 (5.6)***	86.8 (9.0)***	88.2 (11.3)***	85.7 (12.7)***
**SBP**	**p=0.006**	**p<0.001**	**p<0.001**	CON †	116.2 (12.5)	108.1 (13.2)	111.1 (15.1)*	110.0 (15.2)	112.3 (13.5)**	112.7 (13.9)**
(mmHg)				DOB+ ††	126.9 (13.4)	123.6 (5.5)	141.3 (10.7)** **a**	147.9 (7.1)** **ab**	154.2 (10.7)*** **abc**	150.5 (19.4)*** **a**
				DOB- †	112.8 (2.9)	108.3 (8.2)	113.7 (12.4)*	112.2 (8.8)	113.3 (8.8)*	116.5 (7.4)**
				NE †††	108.0 (16.1)	104.9 (16.9)	128.1 (11.5)***	125.3 (16.2)***	126.3 (17.3)***	123.4 (18.9)***
**DBP**	p=0.69	**p<0.001**	**p=0.037**	CON	69.9 (14.3)	61.6 (11.4)	63.6 (12.1)	61.1 (12.3)	61.7 (11.1)	62.0 (11.4)
(mmHg)				DOB+ ††	68.7 (6.6)	62.0 (2.4)	66.5 (2.8)	68.1 (5.2)*	73.3 (5.9)**	73.4 (7.3)**
				DOB-	64.3 (9.1)	59.6 (4.2)	62.7 (5.8)	62.7 (5.1)	65.3 (8.9)	65.4 (7.2)
				NE †	60.6 (11.1)	56.6 (11.8)	68.2 (8.7)**	67.6 (8.8)**	69.1 (13.0)**	66.9 (14.9)*
**TPR**	p=0.94	p=0.76	p=0.15	CON	37.4 (10.6)	29.3 (6.5)	31.2 (6.8)	29.8 (6.8)	28.9 (7.3)	29.9 (9.5)
(mmHg/l/min)				DOB+	34.0 (8.3)	29.0 (4.7)	25.5 (7.5)	26.5 (4.5)	28.1 (4.7)	26.6 (6.3)
				DOB-	35.9 (0.9)	31.4 (6.2)	26.4 (7.6)	27.4 (5.3)	31.6 (6.9)	31.4 (4.4)
				NE	29.4 (4.2)	27.1 (7.6)	30.5 (9.0)	30.3 (12.0)	29.8 (9.4)	28.8 (10.0)
				**Cardiac output and heart rate measures**						
**Q**	p=0.23	**p=0.002**	p=0.22	CON	2386 (486)	2691 (482)	2601 (491)	2665 (549)	2823 (633)	2831 (807)
(ml/min)				DOB+ †	2684 (577)	2897 (465)	3759 (822)*	3636 (480)*	3640 (545)*	3847 (711)**
				DOB- †	2241 (131)	2479 (473)	3179 (835)**	2960 (565)*	2652 (559)	2664 (454)
				NE	2637 (563)	2780 (636)	3156 (1127)	3193 (1037)	3192 (910)	3280 (1027)
**HR**	p=0.61	p=0.11	p=0.09	CON	89.3 (19.0)	98.8 (17.4)	94.6 (15.1)	92.9 (14.3)	91.1 (15.0)	92.4 (14.0)
(bpm)				DOB+	91.1 (14.6)	91.7 (13.2)	101.2 (17.4)	96.2 (6.2)	94.7 (10.6)	101.6 (24.0)
				DOB-	91.9 (14.2)	91.6 (11.7)	101.9 (20.9)	97.1 (13.9)	86.3 (11.8)	82.2 (6.7)
				NE	95.5 (14.8)	96.6 (13.4)	101.7 (25.5)	113.7 (31.9)	108.3 (34.8)	107.2 (35.0)

Supplementary Table 5 *continued*. Experiment 2: systemic and pulmonary hemodynamics

	RM-ANOVA EFFECTS			GROUP	PRE-SCI	POST-SCI				
	time	*group*	*gr × time*		*Baseline*	*30 min (pre-drug)*	*1 h*	*2 h*	*3 h*	*4 h*
**Pulmonary pressure measures**										
**mPAP**	**p=0.017**	**p=0.028**	**p=0.018**	CON	21.1 (4.1)	22.0 (4.6)	22.6 (4.7)	22.6 (4.3)	22.4 (3.6)	22.6 (3.7)
(mmHg)				DOB+	15.4 (2.4)	14.7 (2.2)[a]	14.7 (2.9)[a]	14.9 (2.7)	15.3 (1.8)[a]	15.7 (2.5)[a]
				DOB-	16.7 (2.1)	15.8 (1.4)	16.1 (1.3)	15.8 (1.3)	16.0 (1.5)	16.4 (1.6)
				NE †††	16.5 (3.5)	16.3 (3.3)[a]	18.6 (4.8)*	20.9 (4.9)***	19.2 (5.2)**	18.5 (4.8)*
**PASP** (mmHg)	p=0.45	**p<0.001**	**p=0.017**	CON	31.5 (4.3)	31.6 (5.0)	32.8 (5.3)	33.0 (5.0)	33.0 (4.5)	32.9 (4.8)
				DOB+	27.9 (4.9)	28.2 (4.6)	28.2 (4.6)	30.2 (5.6)	30.0 (5.7)	29.6 (6.0)
				DOB-	27.8 (3.2)	27.5 (3.1)	27.5 (3.1)	27.7 (2.8)	27.6 (2.9)	27.6 (3.1)
				NE ††	25.2 (3.7)	25.3 (4.0)	29.1 (6.9)**	31.5 (7.4)***	30.0 (7.6)**	29.3 (6.7)**

Values are means (SD). CON: control (*n*=8); DOB+: high-dose dobutamine (i.e. ≥2.5 µg/kg/min; *n*=4); DOB-: low-dose dobutamine (i.e. ≤0.5 µg/kg/min; *n*=3); NE: norepinephrine (*n*=7). Statistics are identical to those outlined in Supplementary Table 3. Q: cardiac output. See Supplementary Tables 1 and 2 for additional abbreviations. One-way RM-ANOVA effect of time from 30 min to 4 h post-SCI: †p<0.05; ††p<0.01; †††p<0.001. Between-group comparisons: [a] p<0.05 vs CON; [b] p<0.05 vs DOB - ; [c] p<0.05 vs NE. Within-group comparisons to 30 min (pre-drug): *p<0.05; **p<0.01; ***p<0.001.

Supplementary Table 6. Experiment 2: intraparenchymal spinal cord oxygenation, blood flow and pressure

	RM-ANOVA EFFECTS			GROUP	PRE-SCI			POST-SCI		
	time	*group*	*gr × time*		*Baseline*	*30 min (pre-drug)*	*1 h*	*2 h*	*3 h*	*4 h*
**Spinal cord oxygenation, blood flow and cord pressure at 1.2 cm**										
**SCO_2 1.2cm** (mmHg)	p=0.058	**p<0.001**	**p<0.001**	CON †††	21.9 (13.4)	0.6 (0.1)	0.6 (0.1)	0.6 (0.1)	18.9 (13.4)***	21.9 (13.3)***
				DOB+ †††	22.9 (6.7)	1.7 (1.7)	7.4 (13.2)	10.2 (18.2)	42.5 (10.2)***	47.5 (9.9)***
				DOB-	25.5 (1.2)	1.5 (1.5)	4.8 (7.3)	6.8 (10.7)	17.8 (14.3)	20.8 (11.1)
				NE ††	27.0 (13.0)	0.6 (0.1)	0.9 (0.5)	2.3 (4.1)	26.2 (24.5)*	26.4 (25.3)*
**SCO_2 1.2cm** (%Δ from pre-SCI)	p=0.31	**p<0.001**	**p=0.026**	CON †††	-	-95 (4)	-96 (4)	-96 (3)	-35 (51)**	-8 (63)***
				DOB+ †††	-	-94 (5)	-97 (0)	-96 (1)	89 (27)*** [ab]	112 (27)*** [abc]
				DOB-	-	-94 (7)	-81 (30)	-73 (44)	-54 (16)	-42 (3)*
				NE †††	-	-97 (1)	-95 (5)	-80 (29)	-6 (72)***	-6 (74)***
**SCBF 1.2cm** (a.u.)	**p=0.008**	**p<0.001**	p=0.56	CON †††	178 (66)	66 (23)	86 (47)	70 (30)	255 (45)***	299 (112)***
				DOB+ †††	356 (115)	191 (94)	261 (164)**	328 (275)*** [a]	327 (98)***	364 (119)***
				DOB-	254 (164)	135 (138)	165 (182)	134 (147)	386 (341)	329 (262)
				NE ††	248 (65)	80 (54)	120 (76)	111 (81)	300 (204)***	342 (247)***
**SCBF 1.2cm** (%Δ from pre-SCI)	p=0.125	**p<0.001**	**p=0.003**	CON †††	-	-52 (42)	-51 (37)	-58 (28)	31 (42)***	35 (35)***
				DOB+	-	-47 (19)	-31 (27)	-26 (35)	-14 (37)	-3 (48)
				DOB-	-	-80 (9)	-78 (10)	-82 (11)	23 (77)	6 (53)
				NE †	-	-62 (32)	-47 (37)	-63 (21)	-12 (44) ***	-1 (54) **
**SCP 1.2cm** (mmHg)	p=0.27	**p<0.001**	p=0.20	CON †††	9.5 (3.7)	33.1 (19.8)	32.3 (15.5)	31.7 (11.5)	11.6 (5.4)***	11.5 (5.0)***
				DOB+	6.4 (3.7)	14.7 (6.0)	17.8 (9.7)	24.2 (19.9)	8.9 (1.5)	9.5 (1.9)
				DOB-	6.4 (4.3)	20.1 (15.9)	20.0 (17.1)	16.0 (17.5)	9.2 (7.2)	10.6 (5.9)
				NE †††	8.0 (5.0)	20.2 (8.8)	22.4 (8.4)	26.1 (9.9)	11.1 (3.4) **	10.9 (3.0) **
**SCP 1.2cm** (%Δ from pre-SCI)	p=0.79	**p<0.001**	p=0.59	CON ††	-	345 (372)	338 (351)	348 (378)	20 (28)**	20 (21)**
				DOB+ †	-	236 (302)	306 (384)	399 (434)	98 (154)	133 (227)
				DOB-	-	173 (186)	152 (222)	73 (176)	46 (33)	80 (30)
				NE ††	-	188 (185)	213 (208)	272 (283)	39 (75) *	32 (47) *

Supplementary Table 6 *continued.* Experiment 2: intraparenchymal spinal cord oxygenation, blood flow and pressure

	RM-ANOVA EFFECTS			GROUP	PRE-SCI	POST-SCI				
	time	*group*	*gr × time*		*Baseline*	*30 min (pre-drug)*	*1 h*	*2 h*	*3 h*	*4 h*
**Spinal cord oxygenation, blood flow and cord pressure at 3.2 cm**										
**SCO_2 3.2cm**	**p<0.001**	**p<0.001**	p=0.22	CON †††	25.3 (10.9)	10.3 (9.2)	17.9 (16.4)	23.4 (14.4)	34.7 (13.9)***	42.2 (11.3) ***
(mmHg)				DOB+ †††	35.4 (5.5)	14.4 (5.4)	30.2 (1.3)	55.5 (23.6) [a]	57.2 (11.0)***	57.7 (8.3) ***
				DOB-	22.5 (14.0)	15.4 (5.7)	16.7 (2.7)	19.5 (2.1)	23.1 (16.9) [b]	31.6 (17.7)
				NE †††	39.7 (20.0)	32.3 (12.6) [a]	44.2 (19.9) [a]	49.9 (17.6) [a]	54.8 (17.2)*** [c]	57.5 (16.3)***
**SCO_2 3.2cm**	p=0.21	**p<0.001**	p=0.51	CON ††	-	-47 (42)	-37 (45)	2 (65)	27 (59)*	62 (71)***
(%Δ from				DOB+ †	-	-42 (36)	-8 (16)	22 (18)	35 (40)**	40 (50)**
pre-SCI)				DOB-	-	-8 (56)	-42 (5) [b]	-41 (3)	-46 (58)	-23 (51)
				NE	-	-29 (27)	-4 (40)	13 (42)	22 (32)**	28 (32)***
**SCBF 3.2cm**	p=0.18	p=0.57	p=0.92	CON †	180 (65)	214 (110)	191 (82)	201 (67)	259 (51)	294 (69)*
(a.u.)				DOB+ ††	651 (523)	373 (149)	415 (156)	490 (157)	631 (223)**	660 (232)**
				DOB-	222 (102)	330 (178)	348 (194)	326 (181)	285 (151)	340 (191)
				NE †	390 (267)	958 (1059)	1159 (1381)	1312 (1647)	1152 (1312)	1153 (1064)
**SCBF 3.2cm**	p=0.38	p=0.23	p=0.50	CON ††	-	18 (43)	11 (38)	22 (43)	57 (45)*	81 (60)**
(%Δ from				DOB+ †	-	16 (44)	30 (52)	58 (68)	100 (93)**	96 (105)**
pre-SCI)				DOB-	-	42 (27)	51 (43)	42 (41)	25 (30)	51 (49)
				NE	-	149 (271)	203 (347)	243 (409)	189 (311)	183 (240)
**SCP 3.2cm**	p=0.92	p=0.67	p=0.41	CON †††	14.6 (6.0)	12.6 (5.8)	12.0 (5.6)	12.0 (6.2)	13.4 (5.2)	14.1 (5.4)**
(mmHg)				DOB+	13.0 (0.9)	14.3 (2.1)	13.2 (2.3)	10.4 (6.4)	10.3 (5.4)	11.3 (4.3)
				DOB-	11.2 (0.4)	12.3 (0.78)	11.7 (1.7)	11.5 (1.9)	15.6 (5.7)	15.2 (4.3)
				NE	11.0 (4.6)	15.4 (11.1)	14.7 (10.7)	14.9 (12.7)	13.2 (4.1)	13.2 (2.7)
**SCP 3.2cm**	p=0.30	p=0.89	p=0.75	CON ††	-	-16 (17)	-20 (15)	-22 (17)	-8 (13)**	-2 (16)**
(%Δ from				DOB+	-	10 (15)	2 (16)	-20 (49)	-22 (39)	-14 (30)
pre-SCI)				DOB-	-	10 (7)	4 (16)	3 (18)	40 (56)	36 (43)
				NE	-	51 (131)	47 (129)	53 (154)	34 (59)	34 (26)

Values are means (SD). CON: control (n=8); DOB+: high-dose dobutamine (i.e. ≥2.5 µg/kg/min; n=4); DOB-: low-dose dobutamine (i.e. ≤0.5 µg/kg/min; n=3); NE: norepinephrine (n=7). Statistics are identical to those outlined in Supplementary Table 3. SCO_2: spinal cord oxygenation; SCBF: spinal cord blood flow; SCP: spinal cord pressure; a.u.: arbitrary units. One-way RM-ANOVA effect of time from 30 min to 4 h post-SCI: †p<0.05; ††p<0.01; †††p<0.001. Between-group comparisons: [a] p<0.05 vs CON; [b] p<0.05 vs DOB - ; [c] p<0.05 vs NE. Within-group comparisons to 30 min (pre-drug): *p<0.05; **p<0.01; ***p<0.001.

Supplementary Table 7. Experiment 2: microdialysis measures of spinal cord metabolism

	RM-ANOVA EFFECTS			GROUP	PRE-SCI	POST-SCI				
	time	*group*	*gr × time*		*Baseline*	*30 min (pre-drug)*	*1 hr*	*2 hr*	*3 hr*	*4 hr*
**Lactate**	p=0.48	**p<0.001**	p=0.96	CON (*p=0.07)*	0.36 (0.15)	0.65 (0.19)	0.61 (0.25)	0.57 (0.29)	1.00 (0.40)*	0.88 (0.40)
(mmol)				DOB+ †	0.35 (0.05)	0.64 (0.06)	0.46 (0.12)	0.46 (0.15)	0.85 (0.32)	0.75 (0.28)
				DOB-	0.73 (0.15)	0.86 (0.35)	0.72 (0.00)	0.50 (0.10)	1.39 (0.12)	1.63 (0.13)
				NE ††	0.44 (0.13)	0.71 (0.09)	0.67 (0.10)	0.63 (0.08)	0.92 (0.31)**	0.72 (0.20)
**Pyruvate**	p=0.60	**p<0.001**	p=0.69	CON †††	0.031 (0.008)	0.023 (0.015)	0.018 (0.013)	0.017 (0.011)	0.055 (0.019)***	0.052 (0.020)**
(mmol)				DOB+ ††	0.027 (0.008)	0.031 (0.018)	0.027 (0.017)	0.022 (0.016)	0.045 (0.010)*	0.045 (0.008)*
				DOB-	0.038 (0.009)	0.017 (0.006)	0.010 (0.005)	0.011 (0.009)	0.080 (0.004)	0.106 (0.010)
				NE †††	0.029 (0.009)	0.017 (0.016)	0.015 (0.012)	0.014 (0.010)	0.048 (0.021)***	0.046 (0.018)***
**Lactate/**	p=0.29	**p<0.001**	p=0.61	CON †††	11.9 (3.4)	35.5 (15.1)	50.0 (45.7)	48.2 (45.6)	19.2 (9.2)*	16.6 (4.6)*
**Pyruvate**				DOB+	13.7 (4.1)	32.1 (29.2)	38.4 (49.3)	36.0 (33.6)	19.2 (7.6)	16.2 (3.8)
				DOB-	19.1 (0.9)	59.1 (43.2)	77.8 (35.9)	62.1 (42.1)	17.3 (0.6)	15.4 (0.3)
				NE †††	15.2 (3.9)	59.5 (24.7)	78.1 (53.4)	75.3 (59.4)	21.1 (7.6)*	18.8 (5.6)*
**Glucose**	p=0.20	**p<0.001**	**p=0.009**	CON †††	197 (86)	92 (75)	70 (46)	60 (61)	270 (146)***	295 (140)***
(mmol)				DOB+ †	269 (182)	120 (77)	92 (74)	82 (80)	155 (55)	175 (60)
				DOB-	253 (98)	27 (3)	91 (24)	30 (30)	236 (24)	241 (41)
				NE †††	187 (80)	37 (24)	42 (34)	37 (27)	170 (86)***	172 (88)***
**Glutamate**	p=0.44	**p<0.001**	p=0.29	CON †	0.0019 (0.0015)	0.0070 (0.0031)	0.0026 (0.0017)*	0.0033 (0.0025)	0.0022 (0.0024)*	0.0015 (0.0014)*
(mmol)				DOB+ ††	0.0038 (0.0019)	0.0140 (0.0111)	0.0089 (0.0119)	0.0070 (0.0107)	0.0025 (0.0028)*	0.0017 (0.0016)*
				DOB-	0.0017 (0.0002)	0.0150 (0.0077)	0.0108 (0.0025)	0.0124 (0.0019)	0.0026 (0.0013)	0.0019 (0.0008)
				NE ††	0.0028 (0.0013)	0.0149 (0.0123)	0.0051 (0.0055)	0.0027 (0.0016)**	0.0027 (0.0016)**	0.0021 (0.0008)***
**Glycerol**	p=0.17	**p<0.001**	p=0.35	CON †††	0.012 (0.005)	0.021 (0.003)	0.020 (0.005)	0.022 (0.006)	0.052 (0.020)***	0.047 (0.012)***
(mmol)				DOB+ ††	0.020 (0.008)	0.023 (0.005)	0.022 (0.007)	0.026 (0.009)	0.038 (0.010)**	0.036 (0.008)**
				DOB-	0.022 (0.004)	0.040 (0.020)	0.031 (0.004)	0.030 (0.006)	0.060 (0.007)	0.061 (0.007)
				NE †††	0.017 (0.006)	0.029 (0.007)	0.030 (0.007) [a]	0.034 (0.008) [a]	0.053 (0.022)**	0.057 (0.026)***

Values are means (SD). CON: control (*n*=8); DOB+: high-dose dobutamine (i.e. ≥2.5 μg/kg/min; *n*=4); DOB-: low-dose dobutamine (i.e. ≤0.5 μg/kg/min; *n*=3); NE: norepinephrine (*n*=7). Statistics are identical to those outlined in Supplementary Table 3. Data from DOB- were not included in statistical analyses for microdialysis data due to insufficient sample size (*n*=2). One-way RM-ANOVA effect of time from 30 min to 4 h post-SCI: †p<0.05; ††p<0.01; †††p<0.001. Between-group comparisons: [a] p<0.05 vs CON; [b] p<0.05 vs DOB - ; [c] p<0.05 vs NE. Within-group comparisons to 30 min (pre-drug): *p<0.05; **p<0.01; ***p<0.001.

Supplementary Table 8. Experiment 2: nonparametric data for intraparenchymal oxygenation, blood flow, cord pressure and microdialysis measures

	GROUP	Pre-SCI	Post-SCI				
		Baseline	*30 min (pre-drug)*	*1 h*	*2 h*	*3 h*	*4 h*
			**Spinal cord oxygenation, blood flow and cord pressure at 1.2 cm**				
**SCO_2 1.2cm**	CON						
(mmHg)	DOB+			1.0 (0.7, 14.1)	1.4 (0.7, 19.7)		
	DOB-		0.6 (0.6, 3.3)	0.6 (0.6, 13.2)	0.6 (0.6, 19.3)		
	NE			0.6 (0.6, 1.0)	0.6 (0.6, 0.7)		
**SCO_2 1.2cm**	CON						
(%Δ from	DOB+						
pre-SCI)	DOB-			-98 (-99, -46)	-98 (-99, -22)		
	NE			-96 (-98, -92)	-96 (-97, -56)		
**SCBF 1.2cm**	CON						
(a.u.)	DOB+						
	DOB-			63 (56, 375)	51 (47, 304)		
	NE					236 (177, 289)	228 (166, 487)
**SCBF 1.2cm**	CON		-64 (-74, -47)				
(%Δ from	DOB+						
pre-SCI)	DOB-						
	NE		-78 (-84, -41)				
**SCP 1.2cm**	CON						
(mmHg)	DOB+						
	DOB-			29.8 (0.4, 30.0)			
	NE						
**SCP 1.2cm**	CON		222 (110, 457)	226 (151, 387)	219 (173, 357)	219 (173, 357)	
(%Δ from	DOB+						90 (46, 104)
pre-SCI)	DOB-						
	NE						
			**Spinal cord oxygenation, blood flow and cord pressure at 3.2 cm**				
**SCO_2 3.2cm**	CON						
(%Δ from	DOB+						
pre-SCI)	DOB-						
	NE					38 (9, 42)	
**SCBF 3.2cm**	CON						
(a.u.)	DOB+						
	DOB-						
	NE			421 (172, 3078)	351 (171, 3315)		
**SCBF 3.2cm**	CON			-2 (-17, 33)			
(%Δ from	DOB+						
pre-SCI)	DOB-						
	NE						
**SCP 3.2cm**	CON						
(mmHg)	DOB+						
	DOB-						
	NE		14.5 (7.6, 17.5)	13.2 (8.1, 15.5)	11.8 (7.4, 13.6)		
**SCP 3.2cm**	CON						
(%Δ from	DOB+						
pre-SCI)	DOB-					16 (0, 103)	
	NE		0 (-8, 21)	-6 (-16, 29)	-13 (-18, 30)		

Supplementary Table 8 *continued*. Experiment 2: nonparametric data for intraparenchymal oxygenation, blood flow, cord pressure and microdialysis measures

	GROUP	Pre-SCI	Post-SCI				
		Baseline	*30 min (pre-drug)*	*1 h*	*2 h*	*3 h*	*4 h*
**Microdialysis measures of spinal cord metabolism**							
**Pyruvate**	CON						
(mmol)	DOB+						
	DOB-						
	NE		0.012 (0.009, 0.015)				
**Lactate/**	CON			32.0 (24.4, 51.1)	33.3 (26.2, 50.5)		
**Pyruvate**	DOB+			16.3 (11.6, 65.2)			
	DOB-						
	NE					18.9 (16.1, 23.5)	
**Glucose**	CON						
(mmol)	DOB+		151 (72, 168)				
	DOB-						
	NE						
**Glutamate**	CON	0.0013 (0.0010, 0.0018)				0.0011 (0.0006, 0.0022)	
(mmol)	DOB+			0.0038 (0.0021, 0.1353)	0.0019 (0.0011, 0.0128)		
	DOB-						
	NE			0.0028 (0.0023, 0.0196)	0.0024 (0.0013, 0.0088)		
**Glycerol**	CON						
(mmol)	DOB+						0.040 (0.031, 0.041)
	DOB-						
	NE						

Values for non-parametric data are presented as medians and interquartile ranges (25%, 75%). Only variables with non-parametric data are included. See Supplementary Table 6 for abbreviations. Normalcy was determined using the Shapiro-Wilk test. Microdialysis data are not available for DOB- (insufficient *n*).
