# Supplementary material for: Cardio-centric hemodynamic management improves spinal cord oxygenation and mitigates hemorrhage in acute spinal cord injury
Source: Nat Commun. 2020 Oct 15;11:5209. doi: 10.1038/s41467-020-18905-8 (PMC7562705; doi:10.1038/s41467-020-18905-8)
Supplement: Supplementary file 3 — Reporting Summary [file 41467_2020_18905_MOESM3_ESM.pdf]

nature research

Corresponding author(s): Dr. Christopher West
Last updated by author(s): Aug 26, 2020

Reporting Summary

Nature Research wishes to improve the reproducibility of the work that we publish. This form provides structure for consistency and transparency in reporting. For further information on Nature Research policies, see Authors & Referees and the Editorial Policy Checklist .

Statistics

For all statistical analyses, confirm that the following items are present in the figure legend, table legend, main text, or Methods section.

n/a	Confirmed	
☐	☒	The exact sample size (*n*) for each experimental group/condition, given as a discrete number and unit of measurement
☐	☒	A statement on whether measurements were taken from distinct samples or whether the same sample was measured repeatedly
☐	☒	The statistical test(s) used AND whether they are one- or two-sided<br>*Only common tests should be described solely by name; describe more complex techniques in the Methods section.*
☒	☐	A description of all covariates tested
☐	☒	A description of any assumptions or corrections, such as tests of normality and adjustment for multiple comparisons
☐	☒	A full description of the statistical parameters including central tendency (e.g. means) or other basic estimates (e.g. regression coefficient) AND variation (e.g. standard deviation) or associated estimates of uncertainty (e.g. confidence intervals)
☐	☒	For null hypothesis testing, the test statistic (e.g. *F*, *t*, *r*) with confidence intervals, effect sizes, degrees of freedom and *P* value noted<br>*Give P values as exact values whenever suitable.*
☒	☐	For Bayesian analysis, information on the choice of priors and Markov chain Monte Carlo settings
☒	☐	For hierarchical and complex designs, identification of the appropriate level for tests and full reporting of outcomes
☒	☐	Estimates of effect sizes (e.g. Cohen's *d*, Pearson's *r*), indicating how they were calculated

Our web collection on statistics for biologists contains articles on many of the points above.

Software and code

Policy information about availability of computer code

Data collection	Raw data from the left ventricular (LV) pressure-volume catheter were acquired via an admittance pressure-volume catheter (Sciscence Catheter and ADVantage PV System [ADV500], Transonic Systems Inc). The spinal cord injury impactor device was remotely operated using LabVIEW software (v16.0, National Instruments, Austin, TX). All LV pressure-volume data, pulmonary pressures, cardiac output, blood pressure and heart rate were monitored and acquired using LabChart PRO v8.1.9 (ADInstruments). Measures of cardiac output, as well as LV load-dependent and load-independent variables were made using the LabChart PRO v8.1.9 (ADInstruments) PV Loop Analysis and Cardiac Output modules. Measures of spinal cord oxygenation and blood flow were made in real time using a a multi-parameter probe (NX-BF/OF/E, Oxford Optronix, Oxford, UK) attached to a combined OxyLab/OxyFlo channel monitor (Oxford Optronix OxyLab, Oxford, UK) and interfaced to LabChart Pro software (v8.1.9, ADInstruments, Colorado Springs, CO). Data from spinal cord pressure transducers were acquired digitally with Evolution software (v2.2.0.0 ,FISO Technologies Inc., Harvard Apparatus, Quebec, Canada) and integrated into LabChart Pro. For microdialysis data, dialysates were acquired every 15 minutes and analyzed using ISCUSflex Microdialysis Analyzer (M Dialysis, Stockholm, Sweden). Densitometric analyses of immohistochemistry and histology data were performed using ImageJ (v1.52e, U. S. National Institutes of Health, Bethesda, Maryland, USA)
Data analysis	All statistical analyses were performed using STATISTICA version 13 (TIBCO Software Inc, Palo Alto, CA)

For manuscripts utilizing custom algorithms or software that are central to the research but not yet described in published literature, software must be made available to editors/reviewers. We strongly encourage code deposition in a community repository (e.g. GitHub). See the Nature Research guidelines for submitting code & software for further information.

Data

Policy information about availability of data

All manuscripts must include a data availability statement. This statement should provide the following information, where applicable:

- Accession codes, unique identifiers, or web links for publicly available datasets
- A list of figures that have associated raw data
- A description of any restrictions on data availability

The authors confirm that the data supporting the findings of this study are available within the paper and its Supplementary material.

Field-specific reporting

Please select the one below that is the best fit for your research. If you are not sure, read the appropriate sections before making your selection.

- [x] Life sciences
- [ ] Behavioural & social sciences
- [ ] Ecological, evolutionary & environmental sciences

For a reference copy of the document with all sections, see nature.com/documents/nr-reporting-summary-flat.pdf

Life sciences study design

All studies must disclose on these points even when the disclosure is negative.

Sample size	Prior to this study, there were no published data on left ventricular (LV) contractility (end-systolic elastance, Ees) in a porcine model of spinal cord injury (SCI). However, rodent studies of T2 SCI we have reported a significant mean difference in Ees of 0.67 mmHg/µl with a pooled SD of 0.17 mmHg/µl in T2 SCI vs sham injury (i.e., control). For experiment 1, assuming a power of 0.95 and α of 0.05 we required a minimum of six animals per group to detect significant changes in Ees across four time-points. We chose to include a minimum of seven animals per group to account for any discrepancies in placing the LV-PV catheter and spinal cord probes. For experiment 2 examining the impacts of hemodynamic management, no published data in the porcine model of SCI had reported significant between-group differences in spinal cord oxygenation (SCO2). However, our group had reported a pooled SD of SCO2 (expressed as % of baseline) of 40% in animals receiving vasopressor-based hemodynamic management. With seven animals per group, we were powered to detect a difference of 29% between groups utilizing and SD of 40%, an α of 0.05 and power of 0.95. Experiment 3 was conducted as a proof-of-principle study and thus was not subjected to sample size calculation. The data have not been subjected to statistical analysis nor did we conduct a rigorous sample size calculation. Based on our preliminary data, there is a pooled SD for Ees of 1.08 mmHg/ml in our 12-week survival animals. With a similar number of animals per group to Experiment 2 (i.e. n=7 per group) and an α of 0.05 we would have 95% power to detect a mean difference of 2.07 mmHg/ml between the treatment groups (i.e. DOB vs NE).
Data exclusions	For microdialysis data, we were only able to acquire data from 2 animals in the low-dose dobutamine (DOB-) group, therefore this group was excluded from statistical analyses. However, the data from that group have been included in the Supplemental Tables.
Replication	We did not attempt to replicate our data. We do not believe it would not be ethical to repeat the study with an additional 20+ pigs, and this would also require exceeding amounts of additional operational funds, support staff and operating room time which are not freely available at the Centre for Comparative Medicine (UBC).
Randomization	For Experiment 1, animals all received the same injury with no hemodynamic management, therefore there was no randomization. In Experiments 2 and 3, animals receiving hemodynamic management were allocated either Dobutamine or Norepinephrine treatment utilizing a randomized and counterbalanced design.
Blinding	In Experiment 2, blinding during the experiments was not possible given the drugs administered for hemodynamic management required continuous titration to a given target (i.e. MAP or contractility). The comments throughout data collection in the labchart files regarding the drug levels and multiple measures of Ees and MAP made it impossible to blind for data analysis of primary LV outcomes. However, for all histological and immohistochemistry analyses the observer was blinded to the animal's group/treatment. For Experiment 3, all research staff except for the lead researcher (AW, who administered the treatment) were blinded to the treatment group during the entire duration of the experiment (i.e. injury and treatment day, 12 week survival, end-point measurement day).

Reporting for specific materials, systems and methods

We require information from authors about some types of materials, experimental systems and methods used in many studies. Here, indicate whether each material, system or method listed is relevant to your study. If you are not sure if a list item applies to your research, read the appropriate section before selecting a response.

Materials & experimental systems

n/a	Involved in the study
☐	☒ Antibodies
☒	☐ Eukaryotic cell lines
☒	☐ Palaeontology
☐	☒ Animals and other organisms
☒	☐ Human research participants
☒	☐ Clinical data

Methods

n/a	Involved in the study
☒	☐ ChIP-seq
☒	☐ Flow cytometry
☒	☐ MRI-based neuroimaging

Antibodies

Antibodies used

Rabbit anti-IBA1 (1:1000, Novus NBP2-19019), GFAP-Cy3 conjugate (1:1000, 0.2 ml, Sigma C9205, lot#059M4876V), Alexa Fluor 488 donkey anti-rabbit (abcam ab150073, lot#GR3232538-1)

Validation

IBA-1: Manufacturer suggests dilution between 1:100 and 1:1000. We validated by testing dilutions at 1:100, 1:500, 1:1000 with and without the secondary antibody for a negative control. To date, there are no published data in porcine tissue, however, the manufacturer predicts 100% reactivity with Porcine IBA1 antigens. Manufacturer cites uses in immunohistochemistry-frozen (used in this manuscript), western blot, and immunocytochemistry. Citing articles for immunohistochemistry-frozen use:
1. doi: 10.1371/journal.pone.0228892 (IHC-Fr, Mouse)
2. doi: 10.1371/journal.ppat.1007507 (IHC-Fr, Monkey)

GFAP: Published studies utilizing this product have utilized dilutions between 1:200 and 1:1000. We validated by testing dilutions at 1:100, 1:500, 1:1000 with and with out secondary antibody for a negative control. Manufacturer states reactivity with human, rat, and porcine tissues and applications in immunohistochemistry, immunoblotting, immunolabelling, immunocytochemistry, and immunofluorescence. Citing articles for immunohistochemistry-frozen use:
1. doi: 10.1016/j.apjtm.2017.10.027
2. doi:10.1371/journal.pone.0131059
3. doi:10.1186/1471-2202-12-9
4. doi: 10.1167/iovs.09-4847.
5) doi: 10.1016/j.apjtm.2017.10.027

Animals and other organisms

Policy information about studies involving animals; ARRIVE guidelines recommended for reporting animal research

Laboratory animals

32 female Yucatan minipigs aged 2-3 months (20-25 kg; S&S Farms, Ramona, CA, USA) were acquired and housed in the Centre for Comparative Medicine animal facility for 1-2 weeks prior to surgery. In Experiment 3, 10 of the Yucatan minipigs were survived for 12 weeks with chronic T2 SCI, receiving 24-hour surveillance, continuous analgesia and significant care in the first 14 days. Thereafter, animals were housed in pairs in an enriched environment, with daily monitoring and checks from technicians.

Wild animals

The study did not use wild animals.

Field-collected samples

No field samples were collected in this study

Ethics oversight

All protocols and procedures were compliant with Canadian Council on Animal Care policies, and ethical approval was obtained from the University of British Columbia Animal Care Committee (A16-0311) and United States Department of Defence (IACUC #A16-0311).

Note that full information on the approval of the study protocol must also be provided in the manuscript.
